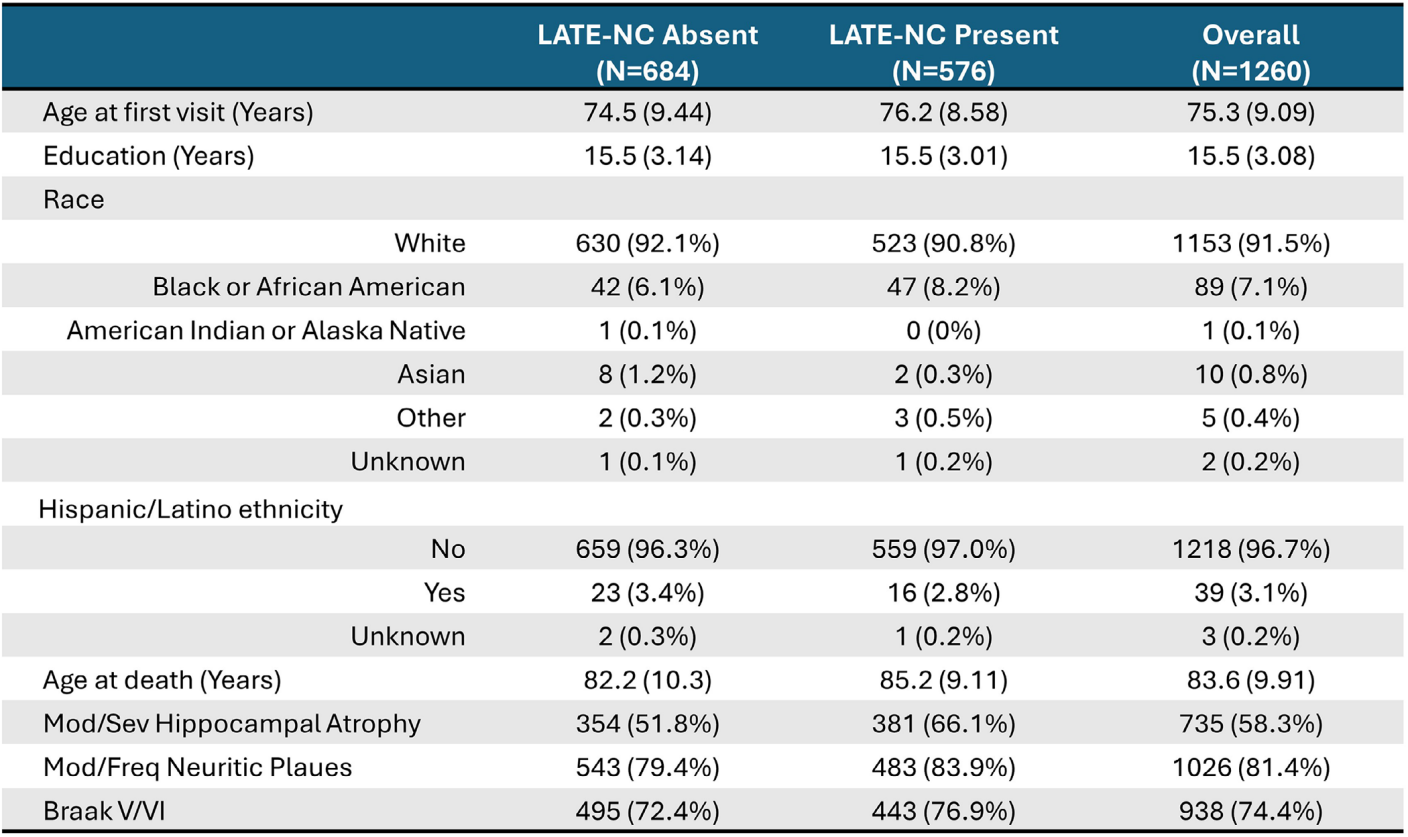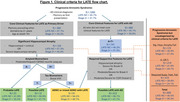# Evaluating the utility of the clinical criteria for LATE using data from NACC

**DOI:** 10.1002/alz70855_106957

**Published:** 2025-12-25

**Authors:** Davis C. Woodworth, Hannah L. Nguyen, Ali Ezzati, Crystal M Glover, María M. M. Corrada, Josh D Grill, Seyed Ahmad Sajjadi

**Affiliations:** ^1^ University of California, Irvine, Irvine, CA, USA

## Abstract

**Background:**

The recent clinical criteria for limbic‐predominant age‐related TDP‐43 encephalopathy (LATE) proposed guidelines for diagnosing LATE during life using clinical assessments and biomarkers. In this study we sought to evaluate the utility of these criteria in identifying LATE neuropathologic change (NC) at autopsy using neuropathology proxies instead of biomarkers.

**Method:**

We used data from participants in the National Alzheimer's Coordinating Center (NACC) including assessments from the last visit before death, autopsy neuropathology, and ages at onset of clinician‐assessed impairment in cognitive domains (memory/language/visuospatial/attention/other). We operationalized the criteria as follows (Figure 1): progressive amnestic syndrome: memory‐first decline with Alzheimer's disease (AD) clinical diagnosis at last visit; core clinical features for LATE: impairment in memory preceding other domains by 2+ years; significant hippocampal atrophy: severe or greater than cortical hippocampal atrophy grossly‐assessed at autopsy (replacing imaging‐based hippocampal atrophy); amyloid‐beta: neuritic plaque score moderate/frequent (replacing amyloid biomarkers); tau: Braak neurofibrillary tangle stage V/VI (replacing tau PET); hippocampal atrophy out of proportion with tau/cognition: severe hippocampal atrophy for Braak VI, moderate/severe hippocampal atrophy for Braak V, or moderate/severe hippocampal atrophy for Clinical Dementia Rating<2 at last visit. We report percentage of any/stage 2+ LATE‐NC and compare rates of LATE‐NC using multilevel logistic regressions accounting for center.

**Result:**

We analyzed data from *N* = 2438 (33.4% LATE‐NC) participants with all necessary data. We classified *N* = 1260 participants (45.7% LATE‐NC) as having progressive amnestic syndrome (Table 1). In those without progressive amnestic syndrome (*N* = 1178) LATE‐NC was present in 20.2%. As shown in Figure 1, few participants met criteria for Probable (*N* = 31, 41.9% LATE‐NC) or Possible (*N* = 12, 50% LATE‐NC) LATE, while many qualified for Possible LATE with AD (*N* = 412, 56.1% LATE‐NC). Rates of LATE‐NC were comparable across LATE groups (though Probable and Possible LATE had few participants), and higher in Possible LATE with AD compared to participants with a progressive amnestic syndrome but otherwise unclassified (*p* <0.001).

**Conclusion:**

In NACC data (which is enriched for ADNC and is limited in racial/ethnic/socioeconomic diversity) and using our operationalization of the clinical criteria for LATE (including neuropathology proxies for biomarkers) Probable LATE and Possible LATE were rare and the rate of LATE‐NC was similar across all LATE groups.